# Understanding support systems for Parkinson's disease management in community settings: A cross‐national qualitative study

**DOI:** 10.1111/hex.13691

**Published:** 2022-12-27

**Authors:** Dia Soilemezi, Ana Palmar‐Santos, M. Victoria Navarta‐Sánchez, Helen C. Roberts, Azucena Pedraz‐Marcos, Anita Haahr, Dorthe Sørensen, Line K. Bragstad, Ellen G. Hjelle, Silje Bjørnsen Haavaag, Mari Carmen Portillo

**Affiliations:** ^1^ Department of Psychology, Faculty of Science and Health University of Portsmouth Portsmouth UK; ^2^ Nursing Department, Faculty of Medicine Universidad Autónoma de Madrid Madrid Spain; ^3^ National Institute for Health Research Applied Research Collaboration Wessex, Long Term Conditions, Southampton UK; ^4^ Academic Geriatric Medicine, Faculty of Medicine University of Southampton Southampton UK; ^5^ Unidad de Investigación en Cuidados y Sistemas de Salud The Carlos III Health Institute (ISCIII) Madrid Spain; ^6^ Grupo de investigación ISCiii Research Network on Chronicity, Primary Care, and Health Promotion (RICAPPS) Tenerife Spain; ^7^ Research Centre for Health and Welfare Technology, Programme for Rehabilitation, VIA University College Aarhus Denmark; ^8^ Nursing and Healthcare, Department of Public Health Aarhus University Aarhus Denmark; ^9^ Department of Nursing Science and Research Center for Habilitation and Rehabilitation Services and Models (CHARM) University of Oslo Oslo Norway; ^10^ Department of Occupational Therapy, Prosthetics and Orthotics Oslo Metropolitan University Oslo Norway; ^11^ Department of Nursing Science University of Oslo Oslo Norway; ^12^ Faculty of Environmental and Life Sciences, School of Health Sciences University of Southampton Southampton UK

**Keywords:** health systems, healthcare utilization, integrated care, long‐term conditions, patient and family carer involvement, professional perspective, resource optimization

## Abstract

**Background:**

Health and social care systems face difficulties in managing multimorbidity, disease burden and complex needs in long‐term conditions such as Parkinson's disease.

**Objective:**

This study aimed to develop a European understanding of how health and social care professionals can collaborate with stakeholders from different organizations and sectors to enhance the management of Parkinson's disease in a community setting by identifying the existing gaps in this process and how people with Parkinson's disease and their family carers could benefit from these partnerships.

**Methods:**

A mixed‐methods sequential study was conducted in Denmark, Norway, Spain and the United Kingdom. The findings from the qualitative phase are presented. Individual semistructured interviews were analysed using Braun's and Clarke's thematic analysis. A meta‐ethnography approach was used to analyse and synthesize cross‐national findings.

**Results:**

A total of 41 healthcare professionals and 39 stakeholders from different disciplines and sectors were interviewed in the four countries. The participants acknowledged a lack of awareness of available resources and poor communication between the different support systems in the management of Parkinson's disease. To promote multiagency collaborations, the participants highlighted the need to organize services along the Parkinson's disease journey, patient involvement and strategic involvement of carers in organizing resources and Parkinson's disease care pathways. According to the participants, the benefits from multiagency partnerships could lead to an enhanced continuity of care and specialized knowledge, mobilization of resources in the community, personalized support and improved access to services.

**Conclusions:**

Policymakers are called upon to create formal structures that facilitate multisectoral collaborations to promote an integrated system of care for the management of Parkinson's disease in the community. To address this challenge, we propose five strategies showing how organizations can work together to optimize the use of resources and enhance the management of Parkinson's disease throughout the illness trajectory.

**Patient or Public Contribution:**

Patient and Public Involvement groups made up of stakeholders, healthcare professionals, patients with Parkinson's disease and family carers participated in the design of the study, the development of the interview guides and the validation of the findings.

## 1 INTRODUCTION

The overall number of people diagnosed with Parkinson's Disease (PD) has been growing progressively globally. In 2019, approximately 8.5 million individuals had received a PD diagnosis.^1^ This estimation is expected to increase to 12 million people in 2050,^2^ indicating that compared to other neurological conditions, PD has the fastest‐growing rate in most countries.

Previous evidence has shown the direct and indirect costs associated with the management of PD, which affects both patients and family carers in relation to hospital admissions, medication, nonmotor symptoms and productivity loss.^3^ The consequences of PD on an individual level may result in the need for continuous support to manage multiple aspects of everyday life, including mobility, work, medication, safety, social life and emotional stability.^3^ In addition, cognitive deterioration in the person with PD (PwPD) may involve a financial burden for the PwPD, the family carer and the health and social care system.^4^, ^5^, ^6^ Thus, support is needed from a long‐term perspective and often increases with the progression of the illness.

Life with PD usually takes place in the community, where PwPD and their family carers have to learn to cope with the PD and its consequences.^7^ Current guidelines contain information regarding medication, symptom management, patient and professional relationships and communication and assessments.^8^ However, with a clear focus on an acute episodic model of care, healthcare services are under pressure and may neglect nonbiomedical consequences of PD (biographical disruptions, negative emotions, strained relationships, nonmotor symptoms and a restriction of meaningful activities), which constitute the most essential burden for patients and families and are the leading causes of hospital re‐admissions and a poor quality of life.^7^, ^9^, ^10^

Furthermore, the existing National Healthcare Systems' personalized self‐care plans and tools fail to capture how people live with and adjust to PD from the PwPD's and the family carers' perspectives. These demands on health and social care systems globally and the limited resources lead to gaps in the care pathways related to manage multimorbidity, disease burden and complex needs and to reach disadvantaged populations, which are understood in this study to be those having immigrant status and/or an ethnic minority background, being older, being socially vulnerable, living with disabilities due to long‐term conditions and being a caregiver.^11^

Self‐management programmes for long‐term conditions are evolving and are now increasingly seen as a collective initiative involving personal networks and other community resources, which go beyond those traditionally known as formal services.^12^ Consequently, this work builds on new understandings of how stronger collaborations between the levels of care and additional support can enhance existing self‐management approaches for PD on a community level ^13^, ^14^ while also reaching disadvantaged areas through more integrated action plans.^11^, ^12^ Previous research has shown that community resources such as voluntary organizations can improve health outcomes through broader forms of support that include the provision of information, physical or social activities, and are better able to reach disadvantaged populations compared with the health and social care services.^15^, ^16^

Furthermore, European recommendations^8^, ^17^ are taking a strategic leap when it comes to placing patients and their families at the centre of decision‐making processes and also regarding the importance of involving various agents in the management of long‐term conditions, including PD. Nevertheless, despite these initiatives, the relationships between agencies are still not clearly established or understood. The lack of awareness of what support is available in the community can lead to an overlap in activities, limited use of community resources and action‐planning gaps.^18^, ^19^, ^20^ Understanding how systems of support for PD management in the community work is essential to enhance the reach of services. Moreover, it is paramount to identify the successful initiatives used by different countries and to learn from established good practices.

In response to the previously mentioned knowledge gaps, the overall aim of this paper is to develop a European understanding of how health and social care professionals can collaborate with stakeholders from different organizations and sectors to enhance the management of PD in the community, and to identify the existing gaps in the collaboration and the potential benefits for PwPD and their family carers.

In particular, the following research questions will be answered:

Q1. How could professionals and stakeholders from different levels of care and sectors work together to improve PD management in the community?

Q2. What are the gaps in the collaboration?

Q3. What could the potential benefits of partnerships for PD management be?

## 2 METHODS

### 2.1 Study design and setting

This article presents the qualitative phase of a sequential mixed‐methods study conducted in Denmark, Norway, Spain and the United Kingdom. This study is part of the OPTIM‐PARK project, which aims to enhance the process of living with PD by designing multisectoral care pathways to optimize the use of community resources across European countries. In this paper, we report findings from the qualitative phase, which is part of the development stage of The UK Medical Research Council framework for developing and evaluating complex interventions.^21^ A strength of this study is the Patient and Public Involvement (PPI) from all countries in different phases to maximize the relevance, applicability and transferability of the findings. The study was reported using the Consolidated criteria for reporting qualitative research (COREQ) (see Supporting Information: File 1).

### 2.2 Participants

A purposeful sampling of health and social care professionals and stakeholders was chosen in each participating country. A total of 40 participants were selected to ensure a broad representation of profiles in each group:

(1) Health professionals from different disciplines that provide support directly or indirectly to PwPD and family carers. The exclusion criteria were an unwillingness to participate in the project or they were not involved in the direct care or support of PwPD.
(2) Stakeholders from different sectors that directly or indirectly impact in the management of PD and the development of care pathways for PD or other long‐term conditions. The exclusion criteria were an unwillingness to participate in the project or a lack of involvement in their role in the strategic planning of community PD care.

**Figure 1:**
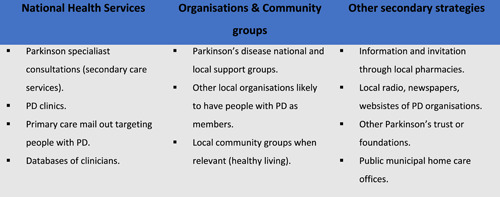
Strategies for recruiting healthcare professionals and stakeholders

Participants were recruited through the strategies shown in Figure 1. Two healthcare professionals and five stakeholders decided not to participate in the interview due to lack of time.

### 2.3 Data collection

Semistructured individual interviews were conducted between April and October 2020 and supported by an interview guide (Table 1), which was developed by all partners (Table 2) and refined by the PPI groups in Spain and the United Kingdom. Interviews initially took place face to face (*n* = 16), although due to the Covid‐19 pandemic, the majority had to be carried out by telephone (*n* = 30) and video conference (*n* = 34). The interviewers (all women) in all countries (Table 2) had extensive experience in conducting in‐depth qualitative interviews.

**Table 1:** Interview schedule for semistructured interviews with stakeholders and health and social care professionals

Topic fields	Questions
Available resources/services/organizations:	What community resources are you aware of for people with Parkinson's and family carers?
	What systems of support are you aware of for people with Parkinson's and family carers?
	What kind/type of support do they provide?
	How did you hear about these resources?
	How can they contribute to more positive living with Parkinson's?
Collaboration between professionals, organizations and levels of care to improve PD management in the community.	Who is responsible in your organization for liaising with other organizations, professionals, policymakers?
	Have you ever, or do you currently, signpost or refer people with Parkinson's or family carers to any of these resources?
	Is there an official pathway within your service to signpost or refer?
	What role do you think different organizations have in the management and daily living of Parkinson's and caring for people with Parkinson's?
	Where do you think the responsibility lies in the provision of care and day‐to‐day living with Parkinson's and caring for people with Parkinson's?
Strategies to maintain these collaborations	What kind of relationship do you have with the different Parkinson's organizations?
	What kinds of relationships do you have with people living with patients and family carers?
	How have you established relationships with these organizations or resources?
	Have any of these relationships changed over time?
The benefits of these collaborations for PD management	What kind of intervention would you find most useful in your current practice/role?
	Do you feel represented in care pathways for Parkinson's?
	How would you define an ideal care pathway in which you have an active role?

**Table 2:** Research partners and Patient and Public Involvement (PPI) representatives involved in this study

	UK	Spain	Norway	Denmark
Research partners	3 members: 1 Psychology, 1 Nursing, 1 Geriatric Medicine	3 members: Nursing	3 members: 2 Occupational therapy, 1 Nursing	2 members: Nursing
PPI	8 members: 1PwPD; 3FCs; 1 user organization; 2 professionals (1 from specialist care, 1 from community care), 1 stakeholder	10 members: 2PwPD; 2FCs; 1user organization; 4 professionals (1 from specialist care, 3 from community care); 1 stakeholder	6 members: 2 PwPD; 1FC; 1 user organization; 2 professionals (1 from specialist care, 1 from community care)	8 members: 1PwPD; 1 FC; 1 user organization; 5 professionals (1 from specialist care, 1 from community care)

Abbreviations: FC, family carer; PwPD, people/person with Parkinson's Disease.

All the participants were also asked to complete a sociodemographic form. The recorded interviews lasted between 32 and 118 min, with an average of 60 min.

### 2.4 Data analysis

**Figure 2:**
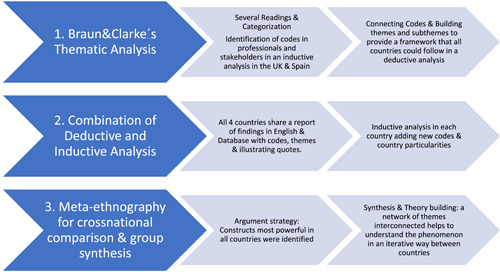
Analysis process

All the interviews were transcribed and analysed following Braun's and Clarke's^22^ thematic analysis combining deductive and inductive approaches (see Figure 2).^23^, ^24^, ^25^, ^26^ The analysis started with an inductive approach with several readings and the categorization of the full transcripts of the professional interviews from Spain and stakeholders interviews from the United Kingdom to provide a framework of analysis connected to the research questions that the other participating countries could follow. All the countries completed their national analyses following the framework provided using a deductive approach and also created additional codes/themes whenever relevant using an inductive approach. An excel database for each analysed group of participants including codes, themes, quotes and a description of the themes was created and shared among all countries.

All the interviews were analysed in the original language of each country, and country‐specific reports were written in English explaining the process followed, and included the findings with quotes for each particular group of participants. A total of 81 themes and 186 subthemes emerged from the analysis across all countries.

Once the country‐specific reports and findings were received, a cross‐national comparison was initiated, which involved multiple readings and discussions across teams towards an analytic synthesis. A meta‐ethnography approach (lines of argument synthesis) was applied,^27^ which helped to interpret and explain the findings across groups and countries, not in an attempt to create generalizations, but to ensure translation from one qualitative case study to another. Using the lines of argument strategy,^27^ the most powerful constructs representing the entire data set from all countries were identified. This led to an agreed conceptual framework that incorporated a network of interconnected themes that are presented in the results and enhanced understanding of the phenomenon under study. This process led to comparative cross‐national synthetic constructs elaborated in the discussion.

### 2.5 Ethical considerations

Following required ethical approval, the participants received a study invitation and were informed of the plans to maintain the participants' confidentiality and anonymity. They all signed an informed consent form. The participants were then allocated a study number and all the names were removed from the analysis and the written national reports.

## 3 RESULTS

In total, 41 healthcare professionals and 39 stakeholders were interviewed across the four countries (Table 3). Most participants were women comprising professionals (85.4%) and stakeholders (79.5%), with an average age of 48.5 and 51.1 years, respectively. In relation to the profile of professionals, many nurses (31.7%), physicians (26.8%) and therapists (34.1%) participated. The role played by the stakeholders in their organization was mainly managerial (48.7%) and direct work with other groups, people with PD or carers (30.1%). Moreover, at least 30% of the participants from each country met the requirement of working actively and directly with vulnerable groups.

**Table 3:** Description of samples in all countries and cross‐national

	Professionals				Stakeholders				Total PROF	Total STH
	UK (*n* = 10)	Spain (*n* = 11)	Norway (*n* = 10)	Denmark (*n* = 10)	UK (*n* = 11)	Spain (*n* = 10)	Norway (*n* = 9)	Denmark (*n* = 9)	41	39
Female	8	10	10	7	10	8	5	8	35	31
Male	2	1	0	3	1	2	4	1	6	8
Age	48 (35–63)	45.5 (31–62)	48.2 (28–67)	52.6 (37–60)	50.6 (38–74)	54 (42–67)	51.7 (38–77)	49.6 (39–59)	48.5 (28–67)	51.5 (38–77)
Years in this position	21.6 (11–41)	22.5 (6–42)	12.9 (5–20)	22.2 (5–35)	9 (0.17–24)	12.9 (2–43)	8.6 (1–16)	7.3 (1.5–20)	19.9 (5–42)	9.5 (0.17–43)
Years working with people with PD level	8.5 (1–29)	18.2 (1–39)	7.5 (1–20)	11.1 (3–20)					14.9 (3–42)	
National					6	4	7	2		19
Regional						5	2	1		8
Local					5	1		4		10
Others								2		2
Professionals' profiles										
Nurse^a^	2	4	4	3					13	
Physician^b^	3	4	1	3					11	
Speech and language therapist	2		1	1					4	
Physiotherapist	2	1	1	2					6	
Occupational therapist		1	2	1					4	
Clinical Psychologist	1								1	
Social worker		1							1	
Training instructor			1						1	
Role in the organization										
Research					2	3				5
Direct work^c^					5	1	2	4		12
Managerial					3	5	6	5		19
Policy						1				1
Other: Strategic lead—service design team					1		1			2

Abbreviation: PD, Parkinson's Disease.

^a^ Nurse: There was variability in the profile of nurses in all countries: PD specialist nurse; Specialized in Neurology; Primary Care Nurse; Nurse (Deprived population).

^b^ Physician: There was variability in their profile in all countries: geriatrician; consultant; Physician—Neurologist; GP (Family doctor).

^c^ Direct Work: usually refers to direct work with: groups; people with PD; carers.

A total of two themes and five categories emerged from the cross‐national analysis and these are presented below (for additional quotes, see Supporting Information: File 2).

### 3.1 Towards more connected systems of support

This theme describes the existing gaps and challenges in the health and social care systems, and the fragmented communication and support in PD management perceived by both professionals and stakeholders. It also covers the benefits that multiagency partnerships could potentially bring to the care and support systems in the four countries, according to the participants.

#### 3.1.1 Staff capacity and training

The stakeholders and professionals identified difficulties in the current systems of support such as an increased workload and overstretched services. The interviewees in the four participating countries discussed the increase in demand and caseload, the reduction in commissioned services and a reduced consultation time, which have all impacted the way the care and support is delivered. As a result, PwPD may call upon alternative support systems such as the family, voluntary organizations and other services to cover the care that the health system cannot provide. Most stakeholders and some professionals in some countries considered that the voluntary organizations were in a better position than the healthcare professionals and had more time to cover informational, social and emotional needs. Both the professionals and stakeholders perceived that the involvement of alternative support systems in PD management was largely dependent on each country's formal system and available funding, the changing political landscape and the individual's commitment to sustain the available support in a specific region, and also that there was a lack of a formal organizational structure and co‐ordination between sectors and organizations.

It's only volunteers that work with these things, so it depends on what resources are available locally. In some places, there is a person or someone very passionate about something that becomes something big there because someone has a lot of energy to do it, and in other places it can be different. (NO‐SH‐003)

Some of the benefits identified by all countries from potential multiagency collaborations were the complementary roles in care and support provision. The professionals (Denmark [DK], Spain [SP], United Kingdom [UK]) and stakeholders (SP, UK, Norway [NO]) highlighted the specific advantages of collaborating with community organizations and the voluntary sector, such as organizing social activities, for example, walking groups, theatre and dance, which could provide peer support, a feeling of belonging and being part of a community, something that the clinical community cannot provide. In any case, identifying other hubs of support in the community was seen as a great opportunity to promote the PwPD's independence from the overstretched health system.

I quite often will suggest just the [name of organisation] website […] actually Parkinson's cafes give people the opportunity to come together once a month, to have a chat, to get some support. (UK‐SH‐008)

In addition, the professionals from all countries commented on the staff's lack of PD specialized clinical skills, from primary care, community services and health centres, which could potentially lead to clinical misjudgements. Many participants indicated the need for education to improve care and support. The participants from Norway and Denmark shared current training opportunities, for example, the Parkinson Net model in Norway, and in Denmark, passionate professionals often educate other professionals about PD symptoms and care.

Health centres have very few patients with PD, so I have actually been out teaching at several of the centres, just to give them the most basic knowledge about PD. (DK‐HCP‐002)

Moreover, in an attempt to foster a multiagency or more connected model of care, it was suggested that all parties could share training resources and best practices to complement each other and ensure continuous professional development. As such, all the agencies could benefit from the existing resources and expertise and avoid duplication, while addressing existing training gaps. The stakeholders and professionals from all of the countries agreed that linking up multidisciplinary and multisectoral teams might facilitate potential continuity of care, better management and knowledge mobilization, which is currently missing. Partnerships were also considered as a path to accessing specialized care that was not formally established in PD care pathways.

You don't see a social worker going with the doctor or nurse for a home visit. When, well, yes, it is important for each one to make their report, okay, but also to see the relationships a bit, right? […] the representatives of the institutions have to negotiate and reach agreements. (SP‐SH‐002)

#### 3.1.2 Awareness of, and communication between, support systems

Health and social care professionals in all four countries acknowledged that they were not always aware of local resources and support that were available as these were constantly changing and very diverse. This made it difficult and frustrating for professionals to navigate the existing resources and to check if the services were still available in their region. An additional issue raised by the interviewees in all countries was that some people could be missing out on the support available because they choose not to be part of the local Parkinson's association.

Our association is of great importance to those who choose to and do sign up; those who choose to participate. Because some choose not to, they can be difficult to get in touch with. And they are probably the ones who need it most, right? (DK‐HCP‐010)

The participants in all of the countries identified that working in silos contributed significantly to the fragmentation of support and communication in PD management. In many cases, due to the bureaucracy and lack of communication between professionals and the different sectors, it has been difficult for clinicians to maintain an overview of the PwPD's history, for example, the admissions, discharges and follow‐ups were not communicated between clinicians.

The community services are not necessarily told if, for example a PwPD falls, and he gets physiotherapy in a private clinic. Then he might tell the physiotherapist, but that information never goes any further. (DK‐SH‐004)

In discussing the potential benefits of working together, the participants in Norway, the United Kingdom and Spain shared examples of past, existing or ideal collaborations, such as when PD nurses had worked closely with consultants and local PD groups, the collaborations between health and social care services and family. According to the participants, reducing the burden of PwPD, maximizing clinical time and thus improving care might be some of the potential benefits from shared information record systems. In addition, sharing communication channels could be cost‐effective and time‐saving. All the participants agreed that effective communication between the levels of care and sectors could create a more connected system, with decision‐making processes involving treatment management being shared between patient, carers and different professionals.

It would be a great advance, to create a truly multidisciplinary team […] we would have a fluid communication that could avoid making the patient dizzy, that the problems are not solved, that ends up in the hospital or in the ER hours and hours, using a resource that is not necessary at that time. (SP‐HCP‐001)

### 3.2 Managing the complexity and support needs of a neurodegenerative disease

This theme captures the complexity of care and support for PwPD and their families to address their increasing vulnerability and social, mental and health needs at various stages of PD. It also illustrates the potential benefits of multiagency, and across organization partnerships throughout the PD journey, such as enhanced support to PwPD and their families through their active engagement.

#### 3.2.1 Timely, meaningful and broader support

Issues concerning the inconsistent support and the lack of long‐term sustainability of the management of PD were discussed by the interviewees in all countries. These inconsistencies could be due to both the geographical location and the complex needs of the PD journey. For example, Norwegian health professionals highlighted that in some municipalities, PwPD did not receive personalized support due to the remote geography of the country.

Some [professionals] have a pure Parkinson nurse position and can be reached all week, from Monday to Friday, some can be reached once a week, while others can only be contacted for a few hours per week. And sadly, this differs greatly from place to place. There is no standardized plan for this. (NO‐SH‐004)

The participants in Denmark and Spain noted the lack of support towards people living with advanced stages of the condition, for example, cognitive decline, and end‐of‐life care.

I would like that there were more resources available for PwPD in the later stages. They are often forgotten. We have offers for all other stages, but in the later stages … arghh … I think something is missing. (DK‐SH‐005)

This postcode ‘lottery’ and lack of standardized provision of support were perceived as potentially creating health inequalities. Professionals from different disciplines and sectors in all countries acknowledged the imperative input to support PwPD in a long‐term perspective, from diagnosis to the advanced stages and criticized the poor management of mental health issues.

We definitely had some patients who desperately needed psychological help […] who were really struggling with coming to terms with the diagnosis, that you know, suffered significant anxiety and depression. (UK‐HCP‐007)

#### 3.2.2 Patient involvement and engagement

Management of the complexity and support needs along the PD journey could not be achieved without patient involvement and their continuous engagement. The lack of patient involvement in the design of services was mentioned by all countries, except the participants from Norway, where PPI in both public and voluntary organizations is well established, and where the PPI representatives are viewed as essential partners. In other countries, PwPD may not be involved in co‐production of care, or treatment plans, and several of the professionals had not considered this.

Many of the participants also experienced a lack of interest in and a low attendance to some of the available support services by PwPD. A barrier to engage in certain resources according to the Norwegian and the UK stakeholders was that the support available was not always flexible and responsive to the individual's particular needs. The professionals from all countries noted that language and location were potential barriers to the attendance to some resources. Professionals also discussed the need for different types of support to appeal to PwPD in different illness stages, from diagnosis to bereavement, and preferences, for example, social groups with many elderly people may not be appealing to younger PwPD, or those who have not accepted their diagnosis may consider it stigmatizing or a forecast of future deterioration.

you can send out a letter to say the department is changing […] feedback from our patient group was what the hell is this? there was a big lesson learning there, in terms of any literature that we are going to send out to patients, we probably need to get patients to read it before we send it out! (UK‐SH‐011)

Building partnerships between disciplines, sectors, PwPD and FC could lead to personalized PD care and ensure continuous engagement with the decision‐making processes. This approach could enable PwPD to be partners and gain sense of control over their PD (self) management.

He is an active patient, that is, you as a health professional will accompany him, you will help him to cope well with his illness, but the one who has to manage his illness is him. (SP‐HCP‐001)

#### 3.2.3 Support to and involvement from carers

Although family carers were generally considered to be a relevant support in managing the complexity of PD, professionals in the United Kingdom and stakeholders in Spain acknowledged that carers were not always involved in designing and implementing care plans with the PwPD, and that carer engagement should start early. All countries acknowledged the need for support to the informal carers, who provide the care and may experience severe stress.

We use the carers; we don't take over the tasks that they have. If carers become exhausted and there is a need for assistance, then that's what we're working towards rather than us starting to relieve carers so that they won't get worn out. (NO‐HCP‐09)

A potential outcome from multiagency partnerships could be proactively offering more support for carers, rather than solely reactively. Carers often lacked the initial knowledge and skills to deal with PD but could be quite resourceful and were proactively seeking help to access information and community/formal resources. Moreover, carers could be signposted to professional services and community resources to prevent burden and stress and offer opportunities for respite time if required.

the family and carers are really active at that diagnosis point and that wasn't really featuring in our service offer … we hadn't realised that family and carers were actually the people doing all the information seeking at that moment. (UK‐SH‐010)

## 4 DISCUSSION

This qualitative study has shown a European understanding of how health and social care professionals and other stakeholders from different agencies and organizations can work together to enhance PD management in the community, what the existing gaps in this process are and how people with PD and family carers could benefit from these partnerships.

The main gaps in PD care identified in our study by the participants were overstretched services, lack of awareness of available resources and support, a limited trained workforce, disjointed services and fragmented communication, inconsistent and limited support, in particular, in mental health issues and advanced stages, and poor patient and carer involvement. Identifying these barriers to multiagency partnerships in PD management is an essential step in planning strategies to address them in European health systems. Recent studies^6^, ^28^, ^29^, ^30^ have also identified three of these barriers, the lack of interdisciplinary management and ongoing support, especially regarding psychological needs, and advanced stages, and the fragmentation of health and social care in other countries.

**Figure 3:**
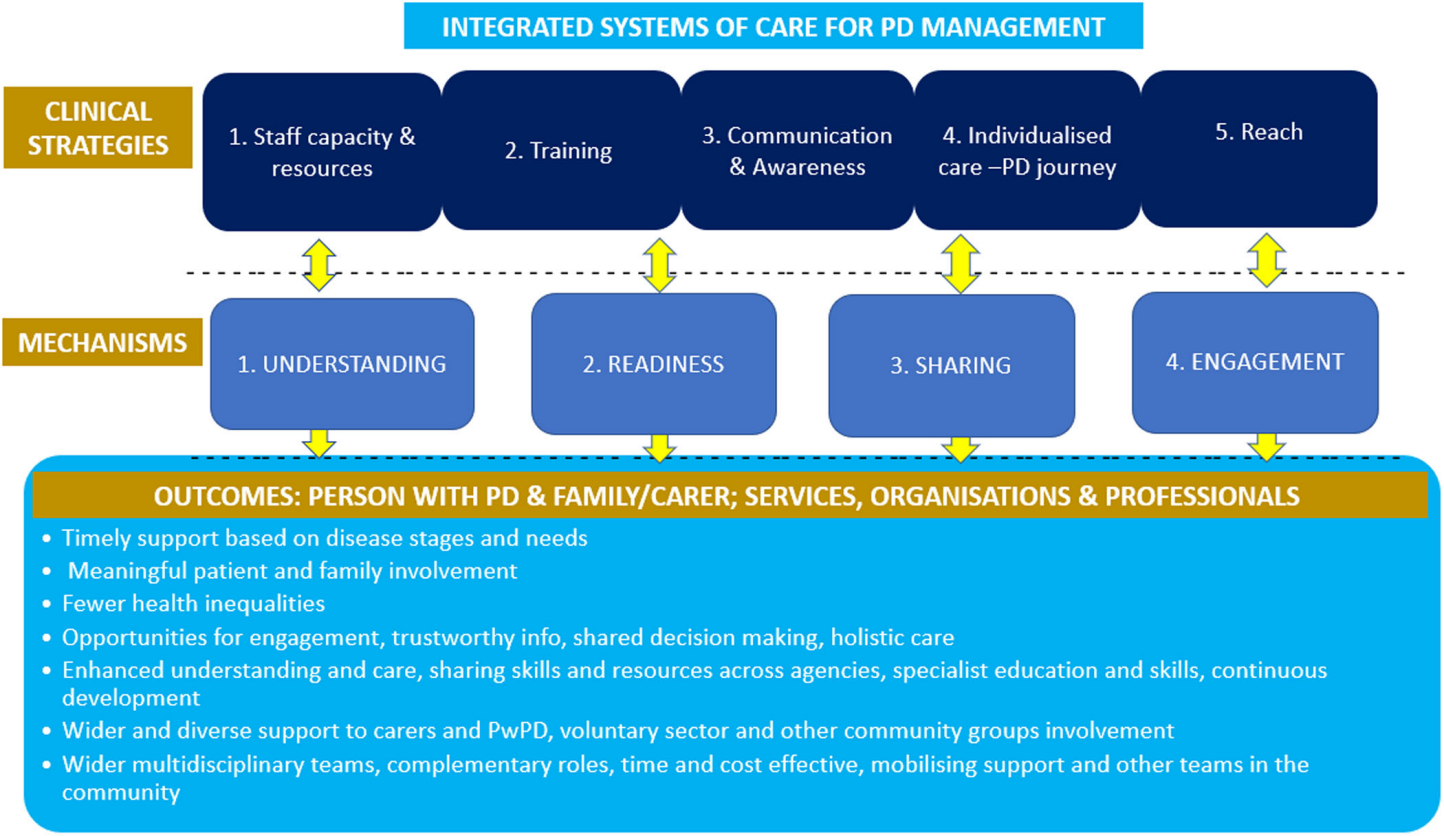
Strategies and mechanisms to sustain a more connected system of care for better PD management in the community. PD, Parkinson's Disease.

To address these gaps, our main findings from health and social care professionals and stakeholders are integrated in Figure 3, which proposes five strategies and four underpinning mechanisms that could make it easier for different organizations to work together to improve PD management in a community setting.

The first strategy is to have the right *staff capacity and resources* to implement integrated systems of care for PD management. This includes the staff having sufficient *training* to obtain *specialist PD skills*, which is the second strategy. To achieve these multiagency collaborations, a macro‐level formal structure to formalize partnerships and care pathways for the PD management in the community might be proposed by policymakers. This could result in shifting priorities towards individualized care and a common vision and agreed agendas. At a meso level, co‐ordination is proposed that will enhance the connections between agencies, levels of care, professionals, the voluntary sector, community organizations, PwPD and family carers and help to navigate and mobilize resources to overcome the staff shortages. These connections could be achieved by an awareness of the roles and resources, specialized training, shared communication systems, complementing expertise and sharing best practice, of the creation of community hubs and identifying PD champions/navigators.

The relevance of creating formal partnerships involving all agencies, that is, the voluntary sector, the community, PWPD and family carers, in PD management has not yet been explored. However, according to the WHO and some comprehensive community‐based programmes,^17^, ^20^, ^31^, ^32^, ^33^ a multisectoral approach has previously been shown to provide benefits in addressing health problems and reducing health inequalities through sharing objectives, pooling resources and optimizing them by avoiding duplication of activities. Furthermore, a multisectoral approach could facilitate two changes: an increase in the number of healthcare professionals who specialize in PD, and community care as the major context for PD management. According to previous studies,^28^, ^34^, ^35^ these changes are essential to improve care for PwPD, especially the most vulnerable, the elderly, to reduce unexpected hospital admissions, carer burden, costs, pressure on the medical system and to enhance the patient's experience and their quality of life. This important change in PD management, from the care delivered mainly in hospitals towards care in the community and in the patients' home, is needed in many countries to achieve a patient‐centred perspective and to address health and nonhealth needs.^28^, ^35^ It is in the community context that PwPD face multiple motor and nonmotor symptoms including cognitive decline^6^ and where PwPD and their family carers face the adjustment process to their new personal, familiar, social and professional roles.^5^, ^36^ Hence, it is important that all health and social care professionals involved in PD management acquire specialist training in PD and an in‐depth knowledge of the role of the different disciplines involved.^28^ The training delivered to multidisciplinary teams in the Dutch ParkinsonNet to increase specialization in PD is an example that has shown improvements in patient outcomes and care costs.^37^

The third strategy is effective *communication between and across* services, organizations, PwPD and their families and *awareness* of what support is available (see Figure 3). The strategy identified in our study is in line with previous international studies that have demonstrated that working with community organizations (beyond the healthcare system) is associated with better health outcomes in people with long‐term conditions.^13^, ^31^ However, there is a gap in these studies as this has not been studied in PD. We propose that individual assessments of social support, from individual social networks and neighbourhoods, and participation in community organizations and the voluntary sector could also bring benefits for PwPD in terms of self‐management and health outcomes. Moreover, improving communication between health and social care professionals, regarding the levels of care, community organizations, the voluntary sector and PwPD and family carers, should be a priority for policymakers to foster multisectoral collaboration and integrated systems of care for PD management.^29^, ^31^

The fourth strategy is *individualized care* along the *PD journey* that promotes timely, meaningful and wider support. The management of PD through this model is paramount to address care fragmentation, poor interdisciplinary care and promote timely access to services and therapies.^28^, ^38^ To promote individualized care throughout the PD journey, it is essential to identify in healthcare a single point of access or a care coordinator, which is an urgent need according to PwPD^28^, ^29^ and long‐term guidelines.^8^ The care coordinator, or single hub, could play a leading role in the assessment process of each person, liaise and work with all health and social care services, the voluntary sector and community organizations and ensure that all referrals to any service or organization start working well for the person.^8^, ^29^

The final strategy is to *reach to PwPD and their families* to ensure meaningful involvement and continuous engagement. We propose, from a micro‐level perspective, that PwPD and their families can become valuable partners that can influence these partnerships and advocate personalized support by their continuous engagement, involvement in clinical decision‐making and the management of their condition and preferred support.

In addition, it is proposed that the PwPD, and their family carers if appropriate, are involved in their needs assessment, as it has been highlighted in other long‐term conditions.^8^ We also propose the need to include the family carers in these assessments to identify any caring, physical and mental health needs.^8^ Fostering self‐management for PwPD is also paramount for a person‐centred approach but also requires ensuring educational and support opportunities.^29^, ^39^

The adoption of this model may result in positive outcomes that are relevant to services, organizations, healthcare professionals, PwPD and their family carers, as described above and shown in Figure 3. Future research should explore the implementation of a multisectoral approach for PD management in a particular context. Future development of tools that help healthcare professionals and stakeholders connect, share resources and optimize communication could also constitute a breakthrough to a more personalized, integrated and cost‐effective PD care.

### 4.1 Limitations

Although we have found important commonalities across country findings, we also acknowledge the existence of cultural differences and the variety of health and social care systems, as well as the use of both inductive and deductive thematic analyses, which could lead to a loss of national findings. However, the wide experience of researchers who undertook all interviews, the involvement of at least two researchers in each country in all analyses, the application of the meta‐ethnography approach (lines of argument synthesis) and the validation from the PPI groups have minimized this.

To our knowledge, this is the first exploratory study across four European countries engaging with a variety of participants to understand how different agencies can collaborate to enhance PD management in a community setting. This study has provided new insights and understanding that could facilitate changes across other countries with established healthcare systems and encourage a more connected system of care in PD and other long‐term conditions.

## 5 CONCLUSIONS

Policymakers are called upon to create formal structures that facilitate multisectoral collaborations between healthcare, social care, community organizations, the voluntary sector and other agents to promote an integrated system of care for PD management in community settings. To address this challenge, five strategies of how different organizations can work together to enhance the management of the different needs throughout the PD journey and the optimization of the resources of the health and social care are proposed.

## AUTHOR CONTRIBUTIONS

**Dia Soilemezi**: Formal analysis; investigation; methodology; validation; visualization; writing – original draft; writing – review & editing. **Ana Palmar‐Santos**: Formal analysis; investigation; methodology; validation; visualization; writing – original draft; writing – review & editing. **M. Victoria Navarta‐Sánchez**: Conceptualization; data curation; formal analysis; funding acquisition; investigation; methodology; validation; visualization; resources; writing – original draft, writing – review & editing. **Helen C. Roberts**: Conceptualization; formal analysis; funding acquisition; investigation; methodology; validation; visualization; writing – review & editing. **Azucena Pedraz‐Marcos**: Formal analysis; Investigation; Methodology; Validation; Visualization; Writing – original draft; Writing – review & editing. **Anita Haahr**: Conceptualization; data curation; formal analysis; funding acquisition; investigation; methodology; validation; visualization; resources; writing – review & editing. **Dorthe Sørensen**: Formal analysis; investigation; methodology; validation; visualization; writing – review & editing. **Line K. Bragstad**: Data curation; formal analysis; investigation; methodology; resources; validation; visualization; writing – review & editing. **Ellen G. Hjelle**: Formal analysis; investigation; methodology; validation; visualization; writing – review & editing. **Silje Bjørnsen Haavaag**: Formal analysis; investigation; methodology; validation; visualization; writing – review & editing. **Mari Carmen Portillo**: Conceptualization; data curation; formal analysis; funding acquisition; investigation; methodology; project administration; resources; validation; visualization; supervision; writing – original draft; writing – review & editing. All authors have contributed to the manuscript substantially and have agreed to the final submitted version. 

## CONFLICT OF INTEREST

The authors declare no conflict of interest.

## ETHICS STATEMENT

This study obtained the ethical approval from the required ethics committees: University of Southampton—IRAS number: 265184; Research Ethics Committee in Hospital Universitario La Princesa number: 3995, CEIm 02/20; Norwegian Centre for Research Data reference number: 986940. Participants gave informed consent before taking part in this study.

## Supporting information

Supporting information.

Supporting information.

## Data Availability

The data that support the findings of this study are available on request from the corresponding author. The data are not publicly available due to privacy or ethical restrictions.
