# Supplementary material for: Understanding support systems for Parkinson's disease management in community settings: A cross‐national qualitative study
Source: Health Expect. 2022 Dec 27;26(2):670–82. doi: 10.1111/hex.13691 (PMC10010098; doi:10.1111/hex.13691)
Supplement: Supplementary file 2 — Supporting information. [file HEX-26--s002.docx]

**Supplementary file 2**. Further quotes to illustrate the findings.

| **Theme 1. Towards more connected systems of support** |
| --- |
| **1.1. Staff capacity and training** |
| *“we need the association to give them rehabilitation, because here... It is very abandoned with resources. In terms of health resources." (SP-SH-001)*  *“I remember that there was one nursing home, where they got an eye-opener. They got this movement-protocol, because the patient had so much involuntary movement, and they just wanted to get a few days [observation] before the patient went home, [to see] how bad it actually was. They had it all wrong, they believed that the patient had too little medication when she jumped off the chair. Therefore, for them it was a revelation and the patient had been walking around like this for about three years or so, being overmedicated, because they had it wrong. It was a super easy thing to solve, but it was a shame for the patient”* *(NO-HCP-002)*    “*… from a patient´s perspective they [voluntary organisations] are more equipped and have the time to spend with patients to discuss the things and also they know all the different other services to signpost to, whereas for us… we might know one or two services to signpost to… we kind of delegate which then frees us up to see other patients* ”*(UK-HCP-009)*    *“We try to have a strong professional organization, divided in specialized teams which gives the possibility to share professional knowledge and get supervision from specialists within neurology”* *(DK-SH-008)* |
| **1.2. Awareness and communication between support systems** |
| *“We do have lots of resources and things that are given to them by the nurse, but that´s part of the reason why I´ve tried to set up this sort of patient passport thing, and I´d like to try and develop that… some sort of diagram that they can follow, that they can see what´s available and what´s coming almost, or when they should be expecting certain things” (UK-HCP-002)*  *“… just try and get us round a table to, again, just try and work out who’s doing what and where we´re covering. You know, that should be fairly easy, but it’s not. So, it´s all these layers of disjointedness” (UK-HCP-008)*  *"I do not see that there is anyone who is coordinating, at least they have not asked us (Primary Care professionals): «With your patients what needs do you have, what barriers do you have, how do you think? That is, to do something in common in a coordinated way (...) to work in common. That is why I find it a bit difficult due to the different areas. And then the social, which clearly comes into play here, with the health, with which they are already two different areas (the social and the health). [...] And then the associations that do everything they can, sometimes even try to do something in common, but that also get lost a lot in our institutional bureaucracy" (SP-HCP-005)*  *“Norway does not even use the same patient record system, they use something different in Trondheim than in Stavanger right?… but when you get things to communicate together, and now a Parkinson registry [for documentation and research] is also established so that data is both self-reported and the neurologist enters data. Imagine if it could communicate with the patient records in a way? (NO-SH-001)*  *“That you can access each other's data [across institutions and sectors], so that I know, for example, that blood pressure has been measured by the GP, so I don't have to waste time taking blood pressure, or that the blood samples have been taken, that would be a huge help ” (DK-HCP-004)* |
| **Theme 2. Managing the complexity and support needs of a neurodegenerative disease** |
| **2.1. Timely, meaningful, and broader support** |
| “*In an ideal world then you would have an interdisciplinary team, with nutritionist, social worker, psychologist, or minister for that matter. For there are many [potentially important professionals]. What we offer [now] is after all very much aimed at the physical*” *(NO-HCP-008)*  “*I think it is the way that [Area] works at the moment, there is a [neighbouring area] part of [area] and a local area part of an area, kind of split into two geographical areas… Whereas area they are discharged which is a disadvantage for someone with Parkinson´s who has got a chronic, progressive disorder”* *(UK-HCP-003)*    *“It is very different, some are attached to the outpatient clinic, and some are attached to the ward. Some have a pure Parkinson nurse position and can be reached all week, from Monday to Friday, some can be reached once a week, while others can only be contacted for a few hours per week. And sadly, this differs greatly from place to place. There is no standardized plan for this”* *(NO-SH-004)*    *“especially mobility barriers, because as soon as things cost money, you can't move, so it's true that Parkinson's doesn't have resources in the neighbourhood. That is to say, it is true, as it is also a neighbourhood that is on the periphery, it doesn't have those resources nearby either, right? So, they are probably not going to have access to specific resources because they are not going to get there” (SP-HCP-005)*  *"In a very good phase of the illness, some maintenance rehabilitation, if anything, maybe once, or twice a week or half hours in hospitals or in centres, it is a small amount (...) When you are very bad, they give you what they can, but at the end (advanced stages) the public resource is limited and they do not offer it, at the private level you have many resources but of course each one has to pay for it". (SP-HCP-007)*    *“…in my experience, one thing is that they need strong strategies to adapt and cope with the physical aspect because it can postpone disease progression. Another thing is the cognitive and social aspects, which we also need to address when designing courses of rehabilitation”* *(DK-SH-002)* |
| **2.2. Patient involvement and engagement** |
| *“Then she was diagnosed with this disease and suddenly found it embarrassing to go out and face the world, and, for example, run into former students. So for her, it was much easier just to stay at home”* *(DK-HCP-010)*    *‘They are older, the disease is very visible and… it is stigmatised a lot and they are very, very isolated. [...] It is a population per se so confined ... So they isolate themselves a lot, they have behavioural problems and others have more emotional problems. They have apathy, depression ... Many are older patients that their children tell them. They say: no, but it is that my father with being able to play the game and such that neither… no, yes neither… Then they don't need much more. He is not a patient ... "(SP-SH-001)*  *“People have to be involved in the decisions they want to make for those years of their life that they are living" (SP-SH-002).*  *“There is a massive social aspect to it, if I am honest about it. I think people feel… people feel two things about [name of organisation]. Some of them find it really, really helpful, they feel they are in a club and they belong and there is people who understand, and other people find it absolutely terrifying because they go there and they are with PD and they are with people with end-stage PD and they are absolutely horrified…"* *(UK-HCP-010)* |
| **2.3. Support to and involvement from carers** |
| *“Suddenly, from one day to the next, your life is turned upside down. The minute you get the diagnosis. Suddenly you need to do certain things, and as a carer you need to act according to a community system and rules, you have never thought of and at the same time, your spouse or relatives gets worse and worse and more demanding, which is really tiring, right?” (DK-SH-006)*    *“One of the huge things that’s come out more recently in our work is that the family and carers are really active at that diagnosis point and that wasn’t really featuring in our service offer… we hadn’t realised that family and carers were actually the people doing all the information seeking at that moment, they’re the ones that are out there really engaging and searching Google and looking on the internet, while the person with Parkinson’s is taking a bit of time to process the diagnosis, they’re having a bit of a time to think but the family member is frantically trying to find out as much as possible.” (UK-SH-010)*  *‘...that was the objective of the multichannel, to establish support networks for carers, right? If someone asks us something very specialised with Alzheimer's, we look for the Alzheimer's association in their area, in their environment, to direct them there..’ (SP-SH-009)* |
